# Agricultural carbon footprints, renewable energy and sustainable development in Asia

**DOI:** 10.1038/s41598-025-17491-3

**Published:** 2025-10-02

**Authors:** Huiran Liu, Yiteng Liu

**Affiliations:** 1 Business School, University of Shanghai for Science and Technology, Shanghai, 200093 China; 2 School of Finance, Shanghai Lixin University of Accounting and Finance, Shanghai, 201209 China

**Keywords:** Agricultural carbon footprints, Environmental sustainability, Climate change mitigation renewable energy, Sustainable development, Energy and society, Environmental economics

## Abstract

There is a growing concern over environmental degradation and climate change in rapidly developing Asian nations. However, little research has been conducted on the impact of agricultural carbon emissions and renewable energy use on sustainable development outcomes in Asia. This research looks at the relationship between agricultural carbon footprints (ACF), renewable energy consumption (RE), and sustainable development (SD) in nine Asian nations from 2000 to 2022. The study employed the Cross-Sectional Augmented Autoregressive Distributed Lag (CS-ARDL), Method of Moments Quantile Regression (MM-QR), and Dumitrescu-Hurlin (DH) panel causality techniques to examine the connection. The findings reveal that the relationships vary significantly across the SD distribution. The findings of DH causality indicate bidirectional causality between agricultural carbon footprints and SD, with economic growth primarily driving agricultural emission patterns rather than the reverse. MMQR results demonstrate that agricultural carbon footprints positively impact SD, with effects strongest at lower sustainability levels but diminishing at higher quantiles, suggesting a nonlinear relationship. Trade openness consistently demonstrates negative relationships with SD across all quantiles, while renewable energy shows positive but statistically insignificant effects. Significant country-level heterogeneity emerges, with China and India demonstrating strong Granger causality from agricultural carbon footprints to SD, while other sampled countries show weaker or insignificant relationships. These findings accentuate the need for contextually appropriate policies that recognize the stage-specific relationship between agricultural practices, renewable energy adoption, and sustainable development outcomes in diverse Asian economies.

## Introduction

The rapid economic expansion has resulted in heightened environmental sensitivity, and significant population pressure have all contributed to highlighting hazards^1^. Agriculture, vital for the economy and food security, has increasingly depended on energy-intensive practices that exacerbate environmental degradation and elevate carbon emissions^2,3^.

Simultaneously, Asia’s energy systems have the significant challenge of reconciling surging demand with sustainable practices, since the region’s persistent dependence on fossil fuels exacerbates pollution and climate issues^1,4^. The geographical and consumption-based greenhouse gas emissions of numerous Asian countries, particularly per capita carbon dioxide emissions, exhibit significant variability across the continent. While several countries fall below the worldwide average, others significantly exceed it. Additionally, numerous Asian nations exert an ecological footprint that significantly exceeds their biocapacity, hence undermining certain aspects of sustainable development^1^.

According to the Global Footprint Network, countries with low biocapacity import greater amounts of biocapacity through trade, resulting in CO₂ emissions that exceed the absorption capacity of their ecosystems.

The relationship between agricultural carbon footprints, renewable energy consumption, and sustainable development in Asia is complex and has significant consequences for the region’s environmental future^5–9^. Agricultural activities in Asia significantly contribute to greenhouse gas (GHG) emissions, with estimates indicating a share that may exceed the commonly referenced 25–30%, reaching as high as 43–44% depending on the specific country and emission source. Emissions primarily result from land-use changes, intensive fertiliser application, methane release from rice paddies, enteric fermentation in livestock, and the fossil fuels that drive mechanised farming. Paddy cultivation and agrochemical use are identified as major emission sources in Asia, attributed to their intensity and scale^4,5^.

In various Asian economies, agriculture constitutes more than 40% of national emissions, highlighting the sector’s significant contribution to regional climate impact^5,6^. Renewable energy serves as a significant mediating factor in this relationship, providing avenues to decrease the carbon intensity of agriculture through the electrification of farm equipment, clean energy-powered irrigation systems, and decentralised renewable solutions for rural agricultural communities. Empirical evidence illustrates this interconnection- Imadojemu et al^1^ found that a 1% increase in renewable energy consumption correlates with a 10.55% decrease in CO₂ emissions in South Asian agricultural systems.

Imandojemu et al.^1^ identified a bidirectional causality in which renewable energy adoption reduces agricultural carbon footprints while simultaneously enhancing agricultural productivity. This relationship supports several sustainable development objectives, notably SDG 7 (affordable and clean energy) and SDG 13 (climate action), while also linking to broader aims of poverty reduction and food security through the enhancement of efficient and resilient agricultural systems. The shift to renewable energy in agriculture is a crucial factor for sustainable development in Asia, effectively tackling climate mitigation, adaptation, and rural development issues in some of the most densely populated and ecologically sensitive areas globally.

Despite progress in the understanding of the environmental effects of agricultural activities, there is still a significant research vacuum on the link between agricultural carbon footprints, renewable energy consumption, and sustainable development in fast growing Asian economies. The empirical literature emphasises the conflict between productivity goals and sustainability objectives by demonstrating that, although the production of renewable energy considerably reduces carbon emissions and ecological footprints across major Asian countries, agricultural value addition usually increases environmental degradation. Thus, our study aims to enhance the science by exploring the interactions among agricultural carbon footprints, the renewable energy consumption, and sustainable development in Asia.

Given their SDG objectives and the partially varied agricultural landscape of Asia, the focus on sustainable development is especially relevant to the region. For instance, while pursuing known renewable energy transitions, China and India together account for over 60% of the agricultural emissions in the region. But since 2000, agricultural exports from countries like Vietnam and Indonesia have grown by more than 400%, raising questions over the carbon footprint of their agricultural explosion. This diversity highlights the need of a comprehensive analytical approach considering many country settings. Thus, we investigate the relationship between sustainable development, renewable energy use, and agricultural carbon footprints between 2000 and 2022 using the Cross-sectional autoregressive distributed lag (CS-ARDL), method of moments quantile regression (MMQR) and the Dumitrescu-Hurlin (DH) panel causality techniques to achieve our objective.

This study makes several important contributions to the literature. First, it extends the discourse on sustainable development in region where her ecological footprint significantly exceeds their biocapacity, by incorporating agricultural carbon footprints and renewable energy as two key determinants of sustainable development, a dimension often overlooked in prior studies. Second, by employing newly moments quantile regression (MM-QR), this study captures heterogeneities in the relationship between sustainable development and its determining factors across different quantiles, providing a better understanding of these dynamics.

Third, the study’s DH Granger causality application offers fresh insights into the causality between economic factors and sustainable development. Lastly, the findings, especially concerning differing impacts, offer essential insights for policymakers aimed at improving sustainability and development in Asia. This finding will improve the understanding of the interactions among these essential variables within Asian economies during a time characterised by significant economic change and environmental issues in the region.

The rest of the paper is laid out as follows: Section "Introduction" details the background. Section "Review of Empirical Review" focuses on extant literature on the link among sustainable development, renewable energy consumption, and agricultural carbon footprints. Section "Methodology and data" details the methodology. Section "Results, Interpretations and Policy Relevance" presents and explores the empirical findings. Major findings and policy implications make up Section "Conclusion and Policy Recommendations".

## Review of empirical review

This empirical review synthesizes the findings from a diverse body of literature that examines various aspects of agricultural greenhouse gas emissions, including mitigation strategies, the relationship between socioeconomic factors and environmental impacts, and the effectiveness of policy interventions. The first standpoint of empirical literatures focuses on agricultural greenhouse gas mitigation strategies. For instance, Aryal et al*.*^10^ examined the effects of crop output on greenhouse gas emissions as well as the main farming-related possibilities for environmental adaptation. The research found that while there are agricultural methods that aid in agriculture’s adjustment to global warming, the institutional frameworks for putting those technological approaches into effect and spreading them have not yet been reinforced.

Aryal^9^ carried out a thorough investigation of methods that can lower greenhouse gases from the agricultural sector by examining supply-side, demand-side, and cross-cutting initiatives. Employing a multifaceted method, the investigation discovered that while there are enormous opportunities to lower greenhouse gas from farming, there are also major obstacles to overcome in order to observe and verify greenhouse gas from supply-side initiatives, transition to environmentally friendly food intake and processing practices, and develop and put in place rules and incentive systems.

Studies provide context for understanding the link between agricultural carbon footprints, renewable energy, and sustainable development in Asia. For example, Asian Development Bank^11^ report highlights biogas digesters for sustainable agriculture, supporting our focus on renewable energy use. Barbier^12^ and Cohen^13^ examine poverty, urbanization, and environmental challenges, aligning with our study’s exploration of socioeconomic drivers. Bouwman et al.^14^ emphasize methane emissions from agriculture, directly relating to our agricultural carbon footprint metric. Bren d’Amour et al.^15^ warn of urban expansion reducing croplands, relevant to land-use pressures. Brock and Taylor^16^ and Brundtland Commission^17^ provide theoretical grounding, while Bloom et al.^18,19^ link health and growth, complementing our sustainability framework.

The study by Cui et al.^20^ revealed a bidirectional relationship between energy use and agricultural emissions, emphasizing the need to integrate energy efficiency policies within the agricultural sector to achieve environmental sustainability and economic growth. On the other hand, Dasgupta et al.^21^ addressed the Environmental Kuznets Curve (EKC), demonstrating that environmental degradation initially rises with economic growth but eventually declines as cleaner technologies and policies are adopted. This framework helps explain how Asian agricultural economies transition toward low-carbon growth as their incomes rise. Furthermore, Degbedji et al.^22^ highlighted the role of institutional quality in promoting green economic growth within the West African Economic and Monetary Union. This implies that strong institutions were shown to enhance environmental governance, a finding relevant to Asia’s agricultural sector where governance capacity varies significantly.

Dernbach^23^ positioned sustainable development as a framework for integrating environmental, social, and economic policies. This foundation is crucial for agricultural policy formulation aimed at reducing carbon footprints and increasing renewable energy adoption. Du et al.^24^ provided evidence from China that targeted agricultural policies can significantly reduce carbon emissions, demonstrating that state-led interventions are essential for climate mitigation in agriculture. Edafe et al. ^25^ explored how large-scale agricultural land investments affect food security in Nigeria. While large-scale agricultural land investments are capable of enhancing production, such investments also risk environmental degradation and social inequality without adequate governance structures. FAO^26^ reported that global agriculture contributes approximately 30% of greenhouse gas (GHG) emissions, primarily through methane and nitrous oxide emissions. This underscores the urgency of adopting renewable energy solutions in the agricultural sector.

Fu et al.^27^ examined the effects of poverty alleviation policies on carbon emissions in China, showing that while these policies reduce poverty, they may inadvertently increase emissions without environmental safeguards. Foley et al.^28^ discussed global consequences of land-use changes, highlighting how agricultural expansion leads to biodiversity loss, environmental degradation, and increased emissions. Gollin et al.^29^ identified large agricultural productivity gaps in developing countries, which result in inefficient land use and higher GHG emissions. Bridging these gaps is key to achieving sustainable agricultural growth. Hanif ^30^ investigated how fossil fuel consumption, energy policies, and urban sprawl contribute to carbon emissions in East Asia and the Pacific. The findings underline the importance of renewable energy transitions to reduce environmental impacts.

He et al.^31^ linked renewable energy use to green economic growth and food security in Sub-Saharan Africa, demonstrating how clean energy adoption addresses both environmental and socioeconomic goals. Imeokparia et al. ^32^ explored the relationship between crude oil exports and poverty reduction in African oil-producing countries, indirectly connecting energy resource management to agricultural sustainability and poverty alleviation. Jayasooriya^33^ identified the agricultural factors most responsible for carbon footprints in Asia, providing essential insights for developing targeted emissions reduction policies.

Jia et al.^34^ examined urban–rural inequality arising from carbon neutrality policies in China. They revealed that while such policies can reduce emissions, they may exacerbate rural–urban disparities without equitable implementation. Jose et al.^35^ proposed a roadmap for agricultural innovation to ensure food security while reducing environmental impacts. Innovation is highlighted as a key driver for sustainable transformation in agricultural systems.

Ju et al.^36^ studied trade openness and foreign direct investment (FDI) impacts on sustainable agriculture in Africa, finding that supportive trade policies foster environmentally friendly practices. Karwacka et al. ^37^ reviewed carbon footprints across the agri-food sector and recommended strategies such as adopting renewable energy and reducing fertilizer use to mitigate environmental damage. Khan et al. ^38^ explored the relationship between poverty, income inequality, and ecological footprints in Asian developing economies, demonstrating that poverty exacerbates environmental degradation. Khan and Yahong ^39^ further showed that rising income inequality directly increases CO₂ emissions and ecological footprints, reinforcing the need for integrated social and environmental policies.

Li et al.^40^ highlighted that promoting clean energy adoption significantly improves food security in Africa, a lesson transferable to Asian agricultural systems. Marques et al.^41^ analyzed food consumption patterns and their links to sustainable development, concluding that shifting diets is essential for reducing agriculture-related emissions. Matthew et al.^42^ examined how information and communication technology (ICT) deployment improves agricultural value chains and reduces inefficiencies, contributing to sustainability. Matthew et al. ^43^ linked agricultural output and carbon emissions to life expectancy in West Africa, emphasizing the health implications of unsustainable agricultural practices.

Mohamed et al.^44^ confirmed that renewable energy in power generation significantly lowers carbon emissions and ecological footprints, supporting the transition toward green energy in agriculture. Osabohien^45^ examined soil technologies and post-harvest losses in Nigeria, highlighting how technological innovation reduces waste and enhances agricultural sustainability. Osabohien and Matthew^46^ connected gender equality (SDG 5) to agricultural productivity and sustainable development, showing that empowering women has direct environmental benefits. Osabohien^47^ focused on environmental stewardship (SDG 15), illustrating how biodiversity conservation intersects with sustainable agricultural practices.

Osabohien et al.^48^ studied the interaction between social protection and environmental policies, finding that well-designed social programs can reduce GHG emissions while improving food security. Osabohien et al.^49^ analyzed agricultural trade and FDI, showing their potential to promote inclusive growth if properly managed. Osabohien et al.^50^ highlighted the importance of agro-financing for boosting food production and reducing poverty in Nigeria. Osabohien et al.^51^ explored the role of social protection in reducing GHG emissions while increasing agricultural productivity. Osabuohien et al.^52^ discussed socioeconomic shocks and their effects on food systems, providing insights into agricultural vulnerabilities.

Oryani et al.^53^ demonstrated that access to renewable energy reduces ecological footprints and improves environmental outcomes, particularly in energy-poor rural regions. Pretty et al.^54^ advocated redesigning agricultural systems for sustainable intensification, calling for systemic changes to meet global food demands without exacerbating climate change. Qi et al.^55^ used advanced modeling techniques to identify drivers of agricultural carbon emissions in China, offering methodological guidance for studies analyzing emissions trends. Ramankutty et al.^56^ linked global agricultural land-use trends to environmental health and food security, underscoring the global dimensions of sustainable agriculture.

Satterthwaite^57^ explained the transition to a predominantly urban world, illustrating how urban growth shapes food systems and agricultural practices. Searchinger et al. ^58^ showed that optimizing land-use changes can significantly mitigate climate change by improving agricultural efficiency. Satterthwaite et al.^59^ explored urbanization’s implications for food production and farming, connecting urban growth with environmental stress. Sehgal and Batool^60^ discussed sustainable agriculture’s critical role in promoting economic development and environmental sustainability in developing nations. Seto et al.^61^ emphasized the sustainability challenges associated with rapid urbanization and its effects on agricultural systems.

Smith et al.^62^ outlined mitigation strategies for agriculture, forestry, and other land uses (AFOLU), providing a comprehensive guide for reducing GHG emissions. Sun et al.^63^ revealed that poverty eradication efforts, while socially beneficial, can lead to higher emissions if not paired with environmental protections. Tilman and Clark^64^ linked dietary changes to environmental sustainability and human health, demonstrating how consumption patterns affect agricultural emissions. UNEP^65^ provided regional assessments of environmental trends, offering macro-level context for sustainability strategies in Asia and the Pacific. Vollrath^66^ connected land distribution to agricultural productivity disparities, showing how unequal land access limits sustainable development.

Whitmee et al.^67^ stressed the need to safeguard planetary health through integrated approaches to food, environment, and health systems. Yoo and Kwak^68^ found that electricity consumption significantly drives economic growth, reinforcing the importance of renewable energy transitions. Yu and Osabohien^69^ explored how education and women’s involvement in agriculture influence human capital outcomes, demonstrating that inclusivity drives sustainable development. Zafeiriou and Azam^70^ investigated CO₂ emissions and economic performance in EU agriculture, offering comparative insights for Asian agricultural policy. Zhang et al.^71^ analyzed spatial and temporal changes in agricultural water footprints, connecting water use to sustainability. Zhen et al.^72^ demonstrated that renewable energy and business density are key drivers of sustainable development.

Anser et al.^73^ examined shadow economies and policy uncertainty, showing how governance gaps influence emissions and sustainability outcomes. Xu et al.^74^ revealed that smart city policies can reduce industrial CO₂ emissions, with indirect benefits for agricultural sustainability. Lin et al.^75^ showed that biodegradable mulching reduces agricultural carbon footprints while boosting crop yields. Teron et al.^76^ studied soil properties and microbial carbon dynamics, adding to the understanding of carbon management in agriculture. Shang and Luo^77^ applied the Tapio Decoupling Principle to urban carbon footprints, offering strategies for reducing agricultural emissions indirectly. Luan et al.^78^ used a system GMM approach to demonstrate that clean energy adoption supports industrialization and sustainable growth. Osabohien et al.^79^ explored the nexus between renewable energy, carbon footprints, natural resource depletion, and economic growth, concluding that renewable energy mitigates environmental degradation.

Osabohien et al.^80^ linked economic growth, climate change, and clean energy in the post-COVID era, emphasizing resilience in sustainable development planning. Guo et al.^81^ examined the role of political institutions and natural resources in sustainable development across Asia, offering insights into governance and resource management. Ramankutty et al.^80^ analyzed global trends in agricultural land use, highlighting rapid expansion driven by population growth, dietary changes, and biofuel demand. The study emphasized how land-use change impacts environmental health through deforestation, biodiversity loss, and greenhouse gas emissions, while also influencing global food security and sustainability strategies.

The above evidence illustrates that literature encompasses several strategies for reducing greenhouse gas emissions from the agriculture sector, such as supply-side, demand-side, and cross-cutting activities. The studies utilize various methodological tools, such as econometric modelling, scenario analysis, and life cycle assessment, to examine the factors and patterns contributing to agricultural greenhouse gas emissions. However, a limitation of the existing literature is that it mostly concentrates on China, with minimal inclusion of various regions or countries. Therefore, broadening the geographical range could offer a deeper understanding of the obstacles and possibilities for reducing agricultural greenhouse gas emissions on a regional and global scale.

## Methodology and data

### Theoretical model specification

This study is premised on ecological modernisation theory (EMT) piloted by Joseph Huber and Martin Jänicke in the 1980s. The ecological modernisation demonstrates that economic development and environmental sustainability can mutually reinforce each other through technological advancement and institutional reform. Unlike radical environmental perspectives that call for de-industrialization or de-growth, EMT argues that modernisation processes can be redirected to attain more ecologically sustainable outcomes.

In view of the foregoing, our employs the EMT theoretical framework to examine the potential for agricultural systems to reduce carbon footprints while maintaining productivity through the incorporation of renewable energy technologies. Agricultural practices reliant on synthetic fertilisers and fossil fuels significantly contribute to greenhouse gas (GHG) emissions, thereby elevating their carbon footprint and threatening ecological balance^2^. The integration of renewable energy in agriculture, such as solar-powered irrigation and biogas systems, serves to decarbonise the sector and enhance energy efficiency.

Empirical findings from Sub-Saharan Africa indicate that agriculture currently contributes to increased emissions; however, the implementation of renewable energy has the potential to mitigate this effect, particularly in low-income areas where conventional energy sources prevail^1^. In densely populated Asian countries, renewable energy significantly reduces ecological footprints, despite rising emissions from agriculture and globalisation, highlighting the essential role of clean energy policies. The environmental Kuznets Curve (EKC) hypothesis posits that environmental degradation initially increases with income growth but subsequently decreases after a certain threshold is reached, particularly with the implementation of sustainable practices such as renewable energy adoption.

Against this backdrop, sustainable development is taken as the function of the agricultural carbon emission, renewable energy consumption, and control variables. This is specified in a multivariate framework as:

1 $$\begin{gathered} \hfill \\ SD_{i,t} = f\left\langle {\left. \begin{gathered} \underbrace {{ACF_{i,t} }}_{{}}\underbrace {{RE_{i,t} }}_{{}} \hfill \\ \underbrace {Main\;actor}_{{}} \hfill \\ \end{gathered} \right|\begin{array}{*{20}c} { \underbrace {{Z_{{n_{i,t} }} }}_{{}}} \\ {\underbrace {{control{\text{var}} iables}}_{{}}} \\ \end{array} } \right\rangle \hfill \\ \hfill \\ \hfill \\ \end{gathered}$$

where ${ACF}_{i,t}$ is agricultural carbon emissions; ${SD}_{i,t}$ is sustainable development; $\underbrace {{RE_{i,t} }}_{{}}$ denotes renewable energy, and the vector of the control variables. The general form of the model could be as follows:

2 $$SD_{i,t} = c_{0} + \beta_{1} ACF_{i,t} , + \underbrace {{RE_{i,t} }}_{{}} + \beta_{2} Z_{i,t} + \mu_{i,t}$$

where Z represent other factors affecting sustainable development (e.g., economic growth (GDP), trade openness (TRADE), and population (POP)), respectively. Equation (2) is re-stated in a linear form as:

3 $${{SD}_{i,t}}={c}_{0}+{\beta }_{1}{ACF}_{i,t}, +{\beta }_{2}{RE}_{i,t}+{\beta }_{3}{GDP}_{i,t}+{\beta }_{4}{TRADE}_{i,t}+{\beta }_{5}{POP}_{i,t}+{\mu }_{i,t}$$

We then log-linearize Eq. 3 as:

4 $${{lnSD}_{i,t}}={c}_{0}+{\beta }_{1}ln{ACF}_{i,t}, +{\beta }_{2}ln{RE}_{i,t}+{\beta }_{3}ln{GDP}_{i,t}+{\beta }_{4}ln{TRADE}_{i,t}+{\beta }_{5}ln{POP}_{i,t}+{\mu }_{i,t}$$

### Data sources and description

The study employs panel data from Asian countries, including Indonesia, Vietnam, India, Malaysia, the Philippines, Bangladesh, China, Thailand, and Mongolia, covering the period from 2000 to 2022. The data were obtained from many sources, including the World Bank, World Development Indicators (WDI), IRENA, the European Commission, and OECD National Accounts.

Our analysis intentionally includes both developing and developed Asian economies to reflect the variety of development stages and policy strategies throughout the region. This methodological selection facilitates a comparative investigation of how nations at varying degrees of economic development address the agriculture-energy-sustainability nexus. The incorporation of advanced economies like the Philippines and Thailand, in conjunction with developing nations such as India and Bangladesh, facilitates the identification of potential technology transfer pathways, opportunities for policy learning, and developmental trajectories that may otherwise be concealed in a more uniform sample.

The criteria for selecting countries covered in this study were predicated on four principal characteristics. Initially, the representativeness of various geographic subregions within Asia. Secondly, disparities in phases of economic growth and levels of agricultural intensification. The variations in renewable energy adoption trajectories, along with the availability and trustworthiness of data throughout the study period, warrant the inclusion of a country in the sample and examine the heterogeneity among the sample countries.

Sustainable Development is dependent variable and is gauged with adjusted net savings, excluding particulate emission damage, in the selected Asia nations. The key independent variable is agricultural carbon footprints, proxy with the Agricultural methane emissions (thousand metric tons of CO_2_ equivalent) following He et al. ^31^ and Osabohien^45^. However, it is both conceptually and empirically appropriate to include GDP per capita, trade openness, and population growth as control variables. As a stand-in for growth in the economy, GDP per capita has an impact on resource use, investment in clean technology, and environmental regulations.

Higher GDP per capita, for example, has been positively correlated with improved environmental sustainability results in Asian settings, suggesting that wealthier economies may be able to both promote growth and alleviate ecological deterioration^3^. Since trade openness influences the spread of green technologies and global environmental standards, it is also a crucial control variable^4^. Based on complementary domestic policies, trade liberalisation can either strengthen or weaken sustainability, according to empirical research. Asian studies have found a favourable correlation between economic growth and sustainability goals.

Additionally, infrastructure and natural resources are strained by population increase, which has a direct impact on environmental sustainability. When assessing the results of sustainable development, it is crucial to account for demographic dynamics because research indicates that growing populations in Asia can have a negative influence on GDP per capita and carbon emissions^6^. By taking demographic pressures, global integration, and economic capacity into consideration, these factors work together to produce a strong and contextually sensitive analytical model that helps isolate the precise effects of agricultural emissions and renewable energy.Table 1.

**Table 1:** Description and measurement.

Variable	Measurement/proxy	Source
Sustainable development (SD)	Adjusted net savings, excluding particulate emission damage (% of GNI)	WDI (2025)
Renewable energy (RE)	Renewable energy consumption (% of total final energy consumption)	WDI (2025), IRENA
Agricultural carbon footprints (ACF)	% of agricultural methane emissions to total	WDI, European Commission
Economic growth (GDP)	GDP per capita (constant 2015 US$)	World Bank WDI (2025)
Trade openness (TRADE)	Trade percentage of GDP	WDI (2025)
Population (POP)	Annual percentage	WDI (2025)

Source: Authors computation, 2025.

### Empirical strategy

The investigation into agricultural carbon footprints and sustainable development dynamics requires rigorous data cleaning methods to guarantee the validity and reliability of empirical results, thereby facilitating the formulation of data-driven, evidence-based policies. Given that the dataset for this study demonstrates panel data characteristics, where the time dimension (T) exceeds the number of countries $(N)$ in each dataset, it is prudent to employ a long panel data estimation procedure for result estimation. The panel estimation procedure comprises six steps. (1) Testing for cross-sectional dependence (CD), (2) assessing slope homogeneity and parameter heterogeneity, (3) estimating panel unit roots, (4) testing for panel cointegration, (5) obtaining panel regression estimates, and (6) conducting Granger causality tests.

### Cross-sectional dependence (CD) and slope homogeneity (SH) testing

Studies have demonstrated that a significant likelihood of cross-sectional dependency in data from several nations, resulting from their socioeconomic activity. Thus, choosing appropriate panel econometric tests for accurate estimation of outcomes is dependent upon testing for cross-sectional dependence (CD) and parameter heterogeneity. Ignoring cross-sectional correlations might result in erroneous estimations and conclusions. This study employed the Pesaran^83^ CD tests to identify panel dependencies, evaluated the heterogeneity assumption and considering the possibility of erroneous estimations and extrapolations resulting from a lack of knowledge of heterogeneity^84^.

### Panel unit root testing

This study employed the Pesaran^83^ CIPS and CADF unit root tests to examine the existence of unit roots in the data. The CIPS and CADF represent second-generation unit root tests designed for heterogeneous panels exhibiting cross-section dependence^85^. The tests have been widely accepted and utilised for determining unit roots in various panel data studies due to their appropriateness^84^.

### Panel cointegration testing

In the present study, we investigated cointegration using three-panel cointegration tests. The cointegration test of Pedroni^86^ comes first. Four of the seven statistics used in the test are based on pooling along the within-dimension (panel v-statistic, panel rho-statistic, panel PP-statistic, and panel ADF-statistic); three are based on pooling along the between-dimension (group rho-statistic, group PP-statistic, and group ADF-statistic). The results shown are meant to prove, against the alternative hypothesis that cointegration exists, the null hypothesis of no cointegration. Whereas the between-dimensional evaluates allow for variation of parameters between panels, the within-dimension tests assume homogeneous autoregressive coefficients across all panels. This flexibility renders Pedroni’s test appropriate for heterogeneous panels.

The Kao^87^ Panel Cointegration test comes in second. This test uses residual-based methods for the null hypothesis of no cointegration in models using panel data. Kao’s method, unlike Pedroni’s test, presumues homogeneous cointegrating vectors over all cross-sections. Five statistics—DF-rho, DF-t, ADF, UDF-rho, and UDF-t—are produced by the test based on the Augmented Dickey-Fuller (ADF) and Dickey-Fuller (DF). The Kao test is particularly useful when dealing with relatively smaller panels and when the assumption of homogeneous cointegrating relationships is reasonable. While Westerlund’s^88^ test was more appropriate due to its suitability in handling cross-sectional dependence (CD) and slope heterogeneity. A key advantage of Westerlund’s approach is that the tests have proven to be reliable in panel cointegration estimation^89^.

### Panel model estimations

Further, to obtain valid and reliable estimates, it is essential to utilise panel data estimators that account for cross-sectional dependence, slope heterogeneity, unit roots, and cointegration within the data. Subsequently, the current study utilised the CS-ARDL, MMQR estimators, and DH causality estimators. This study selected the CS-ARDL and MMQR estimators for their capacity to yield efficient, robust, and reliable results amid slope heterogeneity and cross-sectional dependence^83^. Studies conducted by Anser et al.^6^, have confirmed the suitability of CS-ARDL and MMQR methodologies when addressing cross-sectional dependence, slope heterogeneity, and cointegration in panel studies. The CS-ARDL is structured as:

5 $${SD}_{it}={\omega }_{0}+\sum_{i=0}^{{p}_{e}}{\varnothing }_{i,t}{SD}_{i,t-1}+\sum_{i=0}^{{p}_{x}}{\varphi }_{i,t}{X}_{t-1}+\sum_{i=0}^{{p}_{z}}it{\overline{Z} }_{t-j}+{\mu }_{i,t}$$

where Z = (${\Delta SD}_{it}$, ${X}_{t}$) and 'X' stands for the previously mentioned set of explanatory variables. In the inquiry, the term (*i*) signifies cross-sectional dependence, while the term (*t*) represents the time period. To obtain the average values for both explanatory and dependent variables, ${\overline{Z} }_{t-1}$ is used to resolve cross-sectional dependence based on spillover effects. For each given variable, we include ${p}_{e}, {p}_{x} and {p}_{z}$ in order to determine the lag values.

Alternatively, to capture distributional heterogeneity in the relationship between agricultural carbon footprints and renewable energy at various degrees of sustainable development, the study incorporates the Quantiles via Moments approach, also known as MM-QR with fixed effects, as proposed by Machado and Silva^90^.

MM-QR regression is employed to provide a robust analysis. The MM-QR estimator offers several advantages over conventional panel quantile regression method, if covariates only affect the location and scale functions. Furthermore, this estimator incorporates fixed effects, allowing individual effects to impact the entire distribution. Given these methodologies, the study presents a conditional quantile estimate for a model of location-scale variation:

6 $${\text{QSD}}_{it}\left(\tau |{X}_{it}\right)={\beta }_{0}+{\theta }_{1}{ACF}_{it}+{\theta }_{2}{REN}_{it}+{\mu }_{it}$$

As outlined in Eq. (6), we designate sustainable development, denoted ${\text{SD}}_{it}$, as the dependent variable observed across the sample period. The conditional quantile function at the ${\tau }^{th}$ quantile, denoted as ${\text{QSD}}_{it}\left(\tau |{X}_{it}\right)$, delineates the relationship between sustainable development and explanatory variables. These key variables include agricultural carbon footprints and renewable energy.

The error term, denoted as ${\mu }_{it}$, is assumed to be independently and identically distributed across individual countries i at time t. The residuals, representing the differences between the observed sustainable development and the predicted values based on the explanatory variables, are orthogonal to ${X}_{it}$. Furthermore, these residuals are normalized to satisfy the moment conditions. As articulated in Eq. (7), it can be expressed as follows:

7 $${\text{QSD}}_{it}\left(\tau |{X}_{it}\right)=\left({\propto }_{i}+{\delta }_{i}q\left(\tau \right)\right)+{{X}{\prime}}_{it}\beta +{{Z}{\prime}}_{it}\gamma q\left(\tau \right)$$

Here, expression ${\propto }_{i}\left(\tau \right)={\propto }_{i}+{\delta }_{i}q\left(\tau \right)$, signifies a scalar parameter indicating the quantile-τ fixed effect for individual i. In this framework, Z is a vector of length k that encompasses identified components of X, representing differentiable transformations. Each element ​ is defined as ${Z}_{l}={Z}_{l}\left(X\right),$ where $l = 1, \dots , k.$ Unlike the least-squares fixed effects, the individual effects in this method do not correspond to intercept shifts. The diverse implications of these time-invariant characteristics vary depending on the quantiles of the conditional distribution of the sustainable development. The MM-QR method addresses the optimization problem by estimating the conditional quantile sustainable development function from Eq. (8):

8 $${min}_{q}\sum_{i}\sum_{t}{\rho }_{\tau }\left({\widehat{R}}_{it}-\left({\widehat{\delta }}_{i}+{{Z}{\prime}}_{it}\widehat{Y}\right)q\right)$$

Hence, the standard quantile loss function, denoted as ${\rho }_{\tau }\left(A\right)=\tau -1AI\left\{A\le 0\right\}+\tau AI\left\{A>0\right\}$, is used to measure the deviation from a desired quantile. However, when there is a marginal change in the variable *i*, it affects the parameter of the dependent variable (SD). In this case, i represents the marginal change in the ${\tau }^{th}$ conditional quantile of ${\text{QSD}}_{it}\left(\tau |{X}_{it}\right)$.

### Granger causality test

We estimated the directional causal connection between sustainable development (SD), agricultural carbon footprint (ACF), renewable energy, and control factors across the panels using the Dumitrescu and Hurlin (2012) (D-H) test. The D-H causality test was used given that the method performs well in the presence of slope heterogeneity and cross-sectional dependency.

The D-H test is appropriate for evaluating causalities in panel data by means of bootstrap alternatives. The null hypothesis of no causality forms the foundation of the test. Therefore, considering the variation in development strategies among Asian nations and the necessity to provide accurate and valid results, the study used the following methods to reduce heterogeneity issues in our analysis. To lower heteroscedasticity and raise statistical correctness, first we converted the data into a natural log. Second, we applied two second-generation panel data estimators confirmed to be dependable and fit the features of the data as revealed by the preliminary results. Consistency between the two estimators thus shows the dependability of our findings.

## Results, interpretations and policy relevance

### Pre-estimation results

This sub-section presents the findings of essential pre-estimation tests conducted to assess cross-sectional dependence, stationarity, cointegration, and slope homogeneity among the variables. The results of these diagnostics provide a foundation for reliable estimation, ensuring that the relationships among variables are accurately captured and interpreted.

#### Summary statistics and correlation analysis

With values ranging from almost zero (0.0474) to 37.003 and a mean of 16.197, the descriptive statistics presented in Table 2 expose significant differences in sustainable development (SD) measures across the nine Asian nations for the 2000–2022 period. With a standard deviation of 7.7204, this broad dispersion shows notable differences in sustainability achievements among the countries under sample. With a mean of 57.516, agricultural carbon footprints (ACF) show considerably more variety, ranging from 4.4515 to 94.483, implying different agricultural intensities and practices over these countries. Reflecting different degrees of commitment to and investment in clean energy transitions, renewable energy consumption (RE) likewise shows significant variation (mean of 25.016, range of 1.96 to 60.20).

**Table 2:** Summary of statistics.

	SD	ACF	RE	GDP_PC	TRADE	POP
Mean	16.197	57.516	25.016	3683.3	87.845	1.2356
Maximum	37.003	94.483	60.200	11,555.9	220.40	2.4185
Minimum	0.0471	4.4515	1.9600	620.56	25.993	-0.0131
Std. Dev	7.7204	25.821	14.961	2686.7	48.477	0.5343
Skewness	-0.0756	-0.6165	0.0468	1.2132	0.5792	0.1058
Kurtosis	2.4895	2.0982	2.0798	3.6221	2.3198	2.4762
Observations	207	207	207	207	207	207

Source: Authors’ compilation (2025).

Table 3 presents the findings of the correlation analysis. The findings reveal a positive correlation between renewable energy consumption and sustainable development (0.3772), suggesting that countries investing more in renewable energy tend to achieve better sustainability outcomes. These findings align with theoretical expectations regarding the environmental benefits of clean energy adoption. Similarly, the positive correlation between agricultural carbon footprints and sustainable development (0.3009) presents a paradox, as it indicates that countries with higher agricultural emissions may nonetheless be achieving better overall sustainability scores. This counterintuitive finding might reflect the economic and social development benefits of agricultural productivity that potentially offset environmental costs in the short to medium term, or it could indicate that countries with more developed agricultural sectors have greater capacity to implement sustainable practices.

**Table 3:** Correlation matrix.

	SD	ACF	RE	GDP_PC	TRADE	POP
SD	1					
ACF	0.3009	1				
RE	0.3772	0.3071	1			
GDP_PC	-0.3948	-0.7252	-0.6904	1		
TRADE	-0.4433	-0.2202	-0.5132	0.3837	1	
POP	-0.1900	0.0816	-0.0911	-0.1247	0.3054	1

Source: Authors’ compilation (2024).

The negative correlation between GDP per capita and ACF (-0.7252) further suggests that wealthier economies in the sample have generally managed to reduce the carbon intensity of their agricultural sectors. The negative correlations between sustainable development and both GDP per capita (-0.3948) and trade openness (-0.4433) raise questions about the environmental sustainability of economic development and international trade patterns in these Asian economies. These relationships suggest that economic growth and trade liberalization may have occurred at the expense of sustainable development objectives, potentially due to resource-intensive production methods, weak environmental regulations accompanying rapid economic transformation.

The negative association between population growth and sustainable development (-0.1900), indicates that demographic pressures may pose additional challenges to sustainability efforts. Significantly, the correlation between trade openness and renewable energy consumption (-0.5132) suggests that more open economies in the sample have been slower to adopt renewable energy technologies, perhaps due to competitive pressures in international markets or development models heavily reliant on conventional energy sources.

#### Multicollinearity check

The Variance Inflation Factor (VIF) analysis presented in Table 4 indicate that while some correlation exists among the explanatory variables, multicollinearity does not pose a severe threat to the model’s validity. All VIF values remain below the commonly accepted threshold of 10. GDP per capita exhibits the highest VIF (4.65), suggesting moderate correlation with other predictors—particularly with renewable energy consumption (RE) and agricultural carbon footprints (ACF), as confirmed by the strong negative correlations observed in Table 4.2 (-0.6904 and -0.7252, respectively). This pattern likely reflects the economic development trajectory wherein higher-income countries tend to have both lower agricultural carbon emissions and different renewable energy utilization profiles compared to lower-income counterparts.

**Table 4:** Variance inflation factor.

Variable	VIF	1/VIF
GDP_PC	4.65	0.21514
RE	2.66	0.37552
ACF	2.52	0.396099
TRADE	1.52	0.656182
POP	1.23	0.816198
Mean VIF		2.52

The VIF values for renewable energy (2.66) and agricultural carbon footprints (2.52) indicate some degree of shared variation, while trade openness (1.52) and population growth (1.23) demonstrate relatively higher independence. With a mean VIF of 2.52, the overall level of multicollinearity is acceptable for regression analysis, providing confidence that the estimated relationships between predictors and sustainable development are not substantially distorted by interdependencies among explanatory variables.

#### Testing homogeneity of slopes and cross-sectional dependence

With relatively slight differences mostly related to shared economic, environmental, and geopolitical issues influencing their methods, sustainable development policies across Asian countries show amazing similarities.

Driven by international commitments including the Paris Agreement and the Sustainable Development Goals (SDGs), many of the nations in the region concentrate on including green growth, energy transition, and resource efficiency into their development plans. Reflecting a shared ambition to lower carbon emissions while preserving economic growth, countries including China, India, and South Korea give renewable energy investments, green technology innovation, and low-carbon infrastructure top priority^24^. Consequently, it becomes imperative to investigate and control for cross-sectional dependence to ensure that conclusions drawn from the analysis are robust and not spurious^83^. In our preliminary data analysis, the paper took a cautious approach to assess the homogeneity of slopes, as heterogeneity in slopes, rather than intercepts, is a common concern in standard estimation procedures, as noted by Breitung and Das (2005).

To test for the null hypothesis of no cross-sectional dependence (CD), we employed (Pesaran, 2007a) a cross-sectional dependence test. Additionally, we examined slope homogeneity using the Pesaran and Yamagata^91^ slope homogeneity test. The estimated values of delta tilde (⊿ ®) and adjusted delta tilde (adj⊿ ®) across various probability levels indicated the rejection of the null hypothesis of slope homogeneity at a significance level of 1% (see Table 5).

**Table 5:** Results of cross-sectional dependence and slope homogeneity test.

Panel A: Pesaran (2004) Cross-sectional dependence test		
Variables	CD-Test	abs (Corr.)
Sustainable development (SD)	35.51***	0.709
Agricultural carbon footprint (ACF)	46.75***	0.930
Renewable energy (RE)	35.98***	0.861
Economic growth (GDP)	28.40***	0.987
Population (POP)	12.90***	0.631
Trade Openness	4.12***	0.417

Panel B: Slope Homogeneity Test		
Tests	Statistics	p-value
Pesaran & Yamagata (2008)		
$\widehat{\Delta }$(delta) test	-1.913	0.056 *
$\widehat{\Delta }$(delta) adj. test	-6.049	0.000 ***
Blomquist & Westerlund (2013)		
${\Delta }_{HAC}$	-2.869	0.004 ***
$({\Delta }_{HAC})$ adj	-9.073	0.000 ***

Source: Authors’ compilation (2025).

**p < 0.05; ***p < 0.01.

#### Results of stationary and cointegration tests

Further analysis, as presented in Table 5, confirmed the presence of cross-sectional dependence, with probability values below 1% according to (Pesaran, 2007a). Given the identified cross-sectional dependence and the lack of slope homogeneity, it is imperative to employ estimation procedures that effectively control for these disturbances. Accordingly, we proceeded to estimate second-generation panel stationarity tests, IMP and Pesaran text^83^, which are preferred because of the high precision with cross-sectional dependence in panel unit roots.

These tests are renowned for their precision and ability to address both cross-sectional dependence and homogeneity of slopes in panel data econometrics^92^. By following this rigorous approach, consistent with the recommendations of Phillips and Sul^93^, we aimed to mitigate the risk of obtaining spurious outcomes in our analysis when cross-sectional dependence and slope homogeneity are not adequately accounted for. In Table 6, we presented results from second-generation panel unit root tests, the CIPS test and the CADF test. These tests using CIPS and CADF reveal that most variables, are stationary at first differencing, indicating they are integrated of order 1 (I(1)).

**Table 6:** Results of panel unit root test.

Variables	**CIPS**			**CADF**		
	**Level**	**1**^**st**^** Difference**	**Integration order**	**Level**	**1**^**st**^** Difference**	**Integration order**
SD	-1.019	-3.404 ***	*I*_*1*_	-2.006	-2.421**	*I*_*1*_
ACF	-1.990	-3.975 ***	*I*_*1*_	-2.407	-3.207***	*I*_*1*_
RE	-0.544	-3.132***	*I*_*1*_	-1.074	-2.503**	*I*_*1*_
*GDP*	-1.448	-3.041***	*I*_*1*_	-1.646	-3.041***	*I*_*1*_
*TRADE*	-1.666	-3.615***	*I*_*1*_	-2.124	-2.328**	*I*_*1*_
*POP*	-1.293	-3.109***	*I*_*1*_	-1.846	-3.231***	*I*_*1*_

**p < 0.05; ***p < 0.01; critical values: -2.12, -2.25 and -2.51 for 10%, 5%, and 1% significance level respectively.

Source: Authors’ compilation (2025).

To further analyse long-term cointegrating relationships, we employ the Pedroni^86^, Kao^87^, and Westerlund^88^ cointegration tests. The results of the panel cointegration test in Table 7 indicate a strong presence of long-run equilibrium relationships among the variables. Both the Pedroni^86^ and Kao^87^ tests report several significant statistics at the 1% significance level, suggesting that the variables are cointegrated. In Pedroni’s test, the v-statistic (33.751) and Modified DF t-statistic (9.4405) strongly reject the null hypothesis of no cointegration, with the PP-statistic (-2.4673) and ADF-statistic (4.4698) further supporting the presence of cointegration.

**Table 7:** Results of panel cointegration test.

Pedroni (1999, 2004)		Kao (1999)	Westerlund (2007)
Tests	Statistics	Statistics	
v-statistic	33.751 ***		
Modified DF *t*	9.4405 ***	4.7669 ***	
PP-statistic	-2.4673 ***		
DF *t*		5.8873 ***	
Unadjusted modified DF *t*		-6.4023 ***	
Unadjusted DF *t*		-7.2629 ***	
ADF-statistic	4.4698 ***	6.5340 ***	
Variance ratio			-1.7860**

Note: v: variance; PP: Phillips-Perron; ADF: Augmented Dickie Fuller; DF: Dickie Fuller; *p < 0.1; **p < 0.05; ***p < 0.01.

Source: Authors’ compilation (2025).

Similarly, the Kao test confirms cointegration with significant values for the Modified DF t-statistic (4.7669), DF t-statistic (5.8873), and Augmented DF t-statistic (6.5340). Also, the Westerlund panel cointegration test examines whether a long-run equilibrium relationship exists among variables in a panel dataset. Subsequently, the paper estimates Asia’s sustainable development model using procedures that address the established issues of cross-sectionally dependent.

### Empirical outcomes and discussion

This section elaborates on the empirical results of the investigation. The results of the cross-sectional autoregressive distributed lag (CS-ARDL) are presented in Table 8. The results of the MMQR analysis are shown in Table 9. while Table 10 present results of the D-H Panel Causality tests for Asian countries.

**Table 8:** CS-ARDL.

Variables	Coef	Std. Err	P > z
Short-Run Est			
SD_t-1_	0.5698*	0.2966	0.055
ACF	0.3234	6.7139	0.962
RE	-0.2119	0.7561	0.779
GDP	4.4364	4.6189	0.337
Trade	-0.0359	1.9144	0.985
POP	-2.6251	1.9823	0.185
ECT (-1)	-0.4301	0.2966	0.147
Long-Run Est			
lr_ACF	10.606	7.8880	0.179
lr_GDP	2.9343	10.952	0.789
lr_RE	1.6211	2.3036	0.482
lr_Trade	-1.1913	3.7402	0.750
lr_POP	-1.6439	2.8023	0.557
Diagnostics			
CD test	0.54		
CD test Prob	0.589		
F -stat	19.850**		
R^2^ (MG)	0.84		
Groups	9		

*p < 0.10; **p < 0.05; ***p < 0.01.

**Table 9:** MM-QR Estimation results.

Variables	Coefficients				
	Location	Scale	Q_25	Q_50	Q_75
ACF	0.3939***	-0.4336***	0.6180***	0.1848*	0.0043
	(0.1482)	(0.12859)	(0.2129)	(0.1088)	(0.0880)
RE	0.0526	-0.0545	0.0808	0.0263	0.0036
	(0.0841)	(0.0730)	(0.1170)	(0.0595)	(0.0496)
GDP	-0.0331	-0.1044	0.0208	-0.0834	-0.1269*
	(0.1210)	(0.1050)	(0.16853)	(0.0857)	(0.0714)
Trade	-0.3041***	0.1175	-0.3649**	-0.2474***	-0.1984***
	(0.1165)	(0.1011)	(0.16246)	(0.0827)	(0.0687)
POP	-0.1400	0.0601	-0.1711	-0.1110	-0.0859
	(0.1259)	(0.1092)	(0.1750)	(0.0890)	(0.0742)
Constant	2.6319*	2.6059***	1.2851	3.8887***	4.9736***
	(1.3863)	(1.2024)	(1.9514)	(0.9946)	(0.8202)

*p < 0.10; **p < 0.05; ***p < 0.01; standard errors in parentheses.

**Table 10:** Pairwise Dumitrescu Hurlin Panel Causality Tests.

Null Hypothesis:	W-Stat	Zbar-Stat	Prob
ACF does not homogeneously cause LNSD	5.79835	3.99267	7.00E-05
SD does not homogeneously cause LNACF	7.08004	5.44951	5.00E-08
LNRE does not homogeneously cause LNSD	5.13781	3.24186	0.0012
LNSD does not homogeneously cause LNRE	4.84674	2.91101	0.0036
GDP does not homogeneously cause LNSD	6.99663	5.35470	9.00E-08
SD does not homogeneously cause LNGDP	4.42660	2.43346	0.015
TRADE does not homogeneously cause LNSD	5.53668	3.69524	0.0002
SD does not homogeneously cause LNTRADE	5.62769	3.79869	0.0001
RE does not homogeneously cause LNACF	5.46836	3.61758	0.0003
ACF does not homogeneously cause LNRE	3.56625	1.45553	0.1455
GDP does not homogeneously cause LNACF	14.1386	13.4727	0.0000
ACF does not homogeneously cause LNGDP	4.29985	2.28939	0.0221
TRADE does not homogeneously cause LNACF	2.44616	0.18237	0.8553
ACF does not homogeneously cause LNTRADE	6.61575	4.92177	9.00E-07
GDP does not homogeneously cause LNRE	3.19951	1.03867	0.299
RE does not homogeneously cause LNGDP	1.92030	-0.41535	0.6779
TRADE does not homogeneously cause LNRE	5.10279	3.20206	0.0014
RE does not homogeneously cause LNTRADE	4.65077	2.68826	0.0072
TRADE does not homogeneously cause LNGDP	3.96051	1.90367	0.057
GDP does not homogeneously cause LNTRADE	8.50303	7.06696	2.00E-12

Source: Authors’ compilation (2025).

#### Cross-sectional autoregressive distributed lag (CS-ARDL) results

The results of the Cross-Sectional Autoregressive Distributed Lag (CS-ARDL) are presented and explored in this sub-section. Table 8 shows the dynamic interactions between sustainable development and its underlying factors over the nine Asian nations over 2000–2022. In the short-run analysis, the lagged sustainable development (SD₍ₜ₋₁₎) indicates a positive and significant effect (coefficient = 0.5698, p = 0.055) on current sustainable development levels. This persistence effect implies that prior successes support ongoing progress in sustainability projects, hence generating momentum that transcends short times. This result conforms with the idea of path dependency in sustainable development paths, in which policy continuity and institutional structures are important in preserving sustainability momentum.

The short-run coefficients for agricultural carbon footprints (ACF), renewable energy consumption (RE), GDP per capita, trade openness, and population growth lack statistical significance, however, suggesting that these factors may need time to significantly affect outcomes of sustainable development. These results of a positive but statistically insignificant association between agricultural carbon footprints and sustainable development in the long run resonate with the dynamics observed by Aryal et al.^10^, who noted that although institutional frameworks for implementing these approaches remain underdeveloped even if agricultural methods can help in adaptation to global warming. This implies that governance and implementation difficulties all throughout Asia could limit the possible sustainability advantages of agricultural activities. Our results, however, differ significantly from those of research such as Karwacka et al., who underlined the major environmental impact of the agri-food sector without including possible socioeconomic advantages that might help to support more general sustainable development objectives.

According to long-run estimations from the CS-ARDL model shown in Table 8, agricultural carbon footprints exert positive coefficient (10.606, p = 0.179). This result implies that, by means of economic and social advantages possibly exceeding environmental costs over longer time horizons, agricultural activities may help sustainably development. This result runs counter to some of the studies concentrating just on environmental effects, such Karwacka et al., who underlined the significant ecological influence of the agri-food industry. Nonetheless, it corresponds to the viewpoint presented by Aryal et al.^10^, who acknowledged that, in spite of institutional difficulties in application, agricultural methods can help to adapt to global warming.

The part renewable energy plays in our model—positive but insignificant coefficient—verses more conclusive results in some past research. While our findings imply a more uncertain association between renewable energy especially and sustainable development, Hanif^30^, looking at twelve newly rising East Asian and Pacific countries between 1990 and 2014, discovered that energy consumption clearly affects greenhouse gas emissions. This gap could represent the difference between renewable energy and general energy consumption as well as our emphasis on sustainable development instead of emissions by themselves.

Although not statistically significant at conventional levels (p = 0.147), the error correction term (ECT) coefficient of -0.4301 shows a modest speed of adjustment towards long-run equilibrium. This implies that, indicating a rather effective adjustment mechanism in these Asian countries, about 43% of disequilibrium from the long-run sustainable development pathway is adjusted inside each time period. For example, Oryani et al.^53^ investigated correlations between energy poverty and carbon footprints in South Korea using a dynamic ARDL technique and discovered notable both short-term and long-term adjustment mechanisms.

With a$R2$value of 0.84, the model shows notable explanatory power and confirms that the included variables together account for a good share of variation in results on sustainable development. Though their focus was particularly on the environmental Kuznets curve relationship rather than more general sustainable development indicators, these results notably complement the work of Cui et al.^20^, who used Vector Error Correction Model (VECM) and ARDL approaches to analyse China’s main grain-producing areas and similarly found evidence of adjustment mechanisms in the relationship between economic factors and agricultural emissions.

Table 8's reported diagnostic tests for the CS-ARDL model validate its statistical validity and draw attention to possible sample variability as well. The CD test value of 0.54 (p = 0.589) demonstrates appropriate accounting for cross-sectional dependence, while the significant F-statistic (19.85, p < 0.05) validates overall model validity. Nonetheless, the relatively small individual coefficients point to significant cross-country variability in sustainable development dynamics, much as the country-specific causation studies in Table 11 illustration. This heterogeneity corresponds with findings from Sun et al.^63^, who found that in China, the increase in greenhouse gas emissions could be up to five times greater in developing areas compared to rich ones, so highlighting how differently sustainability challenges and drivers are experienced in different development settings.

Du et al*.*^24^ also observed that the efficacy of policy interventions to lower agricultural emissions varied greatly among Chinese prefectures, with bigger benefits in places with higher agricultural product prices and narrower urban–rural wealth gaps. This implies that local economic, social, and institutional conditions—a complexity that may help to explain the lack of generally significant coefficients in the CS-ARDL results shown in Table 8—probably define the effectiveness of sustainability determinants.

#### Method of moments quantile regression analysis (MM-QR)

Table 9 summarises the Method of Moments Quantile Regression (MMQR) results. The results of the MMQR show diverse effects of explanatory variables over the distribution of sustainable development, therefore providing impressions that would remain hidden in conventional mean-based regression methods. First of all, while trade openness shows a significant negative effect (coefficient = -0.3041, p < 0.01), the location parameter of the MMQR estimates show that agricultural carbon footprints (ACF) exert a strong positive and highly significant influence on sustainable development (coefficient = 0.3939, p These central tendency links imply that, in spite of their environmental impact, agricultural activities help to promote sustainable development outcomes; on the other hand, increased trade integration could perhaps jeopardise sustainability goals.

Especially, the negative and significant (-0.4336, p < 0.01 scale parameter of the MMQR estimate for ACF indicates that the variability in sustainable development outcomes reduces with higher agricultural carbon footprints—suggesting that more agriculturally intensive economies may exhibit more predictable, if not necessarily optimal, sustainability patterns. Moreover, agricultural carbon footprints show especially significant positive impacts on sustainable development (coefficient = 0.6180, p < 0.01) at the bottom quantile (Q_25) of the Table 9, which represents nations with rather lower sustainability performance.

This strong correlation implies that, despite their carbon consequences, agricultural activities benefit general sustainability for nations in earlier phases of sustainable development most likely by means of contributions to food security, rural livelihoods, and economic stability. With a positive but statistically weak coefficient (0.0808), renewable energy consumption indicates that nations with poorer sustainability performance could have implementation difficulties or scale restrictions preventing them from entirely realising the sustainability benefits of renewable energy adoption. At this quantile (-0.3649, p = 0.05), trade openness exhibits a notable negative relationship with sustainable development, suggesting that nations with less developed sustainability frameworks may be particularly vulnerable to possible negative effects of international trade integration, presumably due to weaker environmental governance or greater exposure to resource-extractive trade patterns.

Table 9 shows changes in variable relationships for nations at the median quantile (Q50) of sustainable development performance. Agricultural carbon footprints show a positive but declining impact on sustainable development (coefficient = 0.1848, p < 0.10), therefore implying declining marginal rewards to agricultural carbon emissions as nations move along the sustainability spectrum. Though with a smaller coefficient magnitude than at the lower quantile, trade openness continues to show a notable negative relationship with sustainable development (-0.2474, p < 0.01), suggesting that nations with moderate sustainability performance may have developed some institutional capacity to mitigate perhaps negative trade effects. At this quantile, GDP per capita has a negative but negligible correlation with sustainable development, perhaps reflecting environmental Kuznets curve dynamics whereby economic growth first damages environmental quality before finally supporting improvements. The continuous insignificance of renewable energy coefficients across quantiles begs issues concerning implementation difficulties, policy consistency, or scale restrictions that would prohibit these Asian economies from fully using renewable energy for sustainability improvements.

Moreover, the link between agricultural carbon footprints and sustainable development becomes statistically negligible (coefficient = 0.0043) at the top quantile (Q_75), therefore representing the sustainability leaders among the sampled nations. This change implies that for high-performance nations, additional increases in agricultural carbon emissions no longer translate into better outcomes for sustainable development; rather, this suggests a threshold effect whereby agricultural productivity benefits have been mainly realised and further intensification yields declining returns for more general sustainability goals.

At this upper quantile (-0.1269, p < 0.10), GDP per capita shows a notable negative relationship with sustainable development, implying that for leaders in sustainability, ongoing economic growth may provide difficulties in maintaining environmental quality without suitable protections and decoupling mechanisms. Though with a continuous reduction in coefficient magnitude, indicating that high-performance countries have developed greater resilience to possible adverse trade effects, though not completely eliminated, trade openness maintains its significant negative association with sustainable development (-0.1984, p < 0.01.

At lower quantiles, the strong positive correlation between agricultural carbon footprints and sustainable development, however, fits dynamics found by Aryal et al*.*^10^, who noted that although agricultural activity generates greenhouse gas emissions, it also offers necessary adaptation benefits. But the current study results extend this intuition by showing how this link differs methodically along the distribution of sustainable development—a informed point not well noted in earlier research. The environmental Kuznets curve relationship found in China’s grain-producing areas, where economic development first raised agricultural emissions before reaching a turning point, resonates with the declining positive effect of agricultural carbon footprints across higher quantiles of sustainable development.

Although the continuously low coefficients for renewable energy across all quantiles run counter to some earlier research stressing the good environmental effects of this energy source. This discrepancy probably reflects our emphasis on overall sustainable development instead of more limited environmental measurements as well as possible implementation difficulties pointed out by Aryal^9^, who noted "major obstacles to overcome in order to observe and verify greenhouse gas from supply-side initiatives. Our results imply that despite theoretical advantages, adoption of renewable energy sources in these Asian countries has not yet resulted in appreciable sustainable development improvements—probably due to scale constraints, implementation challenges, or policy inconsistencies underlined in the literature review.

Moreover, the negative correlation between trade openness and sustainable development over all quantiles offers a gloomier picture than some earlier research. This result supports the observations of Khan and Yahong^39^ that more wealth inequality—often connected with some forms of trade liberalization—helps to cause environmental degradation in Asian rising countries. Consistent with Du et al.'s assessment that local economic conditions greatly affect the efficacy of sustainability policies, the declining size of negative trade effects across higher quantiles also suggests that more sustainably developed economies have greater capacity to mitigate adverse trade impacts.

Unlike past research, the quantile-specific GDP effects become notably negative only at the highest sustainability quantile. This trend supports Hanif’s^30^ observation that economic growth greatly raises greenhouse gas emissions in developing East Asian and Pacific countries, but it also implies that this link may rely on the current sustainability performance of states. In line with Qi et al.'s^56^ finding that agricultural emissions patterns in Chinese provinces are much influenced by economic factors, our data also add the crucial observation that these interactions vary methodically along the spectrum of sustainable development.

Furthermore, the heterogeneous effects across quantiles in the current study significantly support Sun et al.^63^ finding that sustainability challenges vary greatly depending on the development framework; greenhouse gas emissions increase maybe five times more in developing areas than in rich ones. Our quantile method formalises this heterogeneity by showing how important sustainability factors behave differently at different places in the sustainable development distribution. This viewpoint reveals how the relevance of agricultural emission drivers probably changes as nations progress along their sustainable development paths, therefore extending Jayasooriya’s^33^ study of these drivers across 46 Asian countries.

#### Causality analysis

The results of the Dumitrescu-Hurlin panel causality test are presented in Table 10. The results demonstrate bidirectional causality between sustainable development (SD) and agricultural carbon footprints (ACF), with highly significant test statistics in both directions (p < 0.0001) in the nine Asian economies. This mutual causality suggests that agricultural activities influence sustainability outcomes through environmental impacts, economic contributions, and social dimensions, while sustainability performance simultaneously shapes agricultural practices through policy frameworks, resource constraints, and changing consumption patterns.

Similar bidirectional causality emerges between sustainable development and renewable energy consumption (RE), with significant causal flows in both directions $(p < 0.01)$, indicating that renewable energy adoption both drives and responds to broader sustainability initiatives. The robust bidirectional causality between sustainable development and GDP per capita $(p < 0.0001$ from GDP to SD; $p < 0.05$ from SD to GDP) underscores the relationship between economic development and sustainability, where economic growth influences environmental and social outcomes while sustainability performance increasingly constrains and shapes economic trajectories through resource limitations, regulatory environments, and evolving market preferences.

The strong bidirectional relationship aligns with Cui et al.'s^20^ investigation of China’s major grain-planting regions, which employed similar methodological approaches—including Granger Causality Tests based on Vector Error Correction Models—and found significant bidirectional relationships between economic characteristics and agricultural greenhouse gas emissions. However, our finding extends beyond China to a broader Asian context and specifically addresses sustainable development rather than merely emissions, providing a more comprehensive sustainability perspective. This bidirectional causality also complements Aryal et al.'s^10^ observation that "while there are agricultural methods that aid in agriculture’s adjustment to global warming, the institutional frameworks for putting those technological approaches into effect and spreading them have not yet been reinforced"—suggesting that feedback loops between agricultural practices and sustainability outcomes are moderated by institutional capacity.

Table 10 also demonstrates a strong unidirectional causality flows from GDP per capita to agricultural carbon footprints (p < 0.0001), with no significant causality in the reverse direction, suggesting that economic development substantially transforms agricultural practices and their associated carbon profiles—potentially through mechanization, intensification, or structural shifts in production systems. Agricultural carbon footprints and trade openness exhibit an asymmetric causal relationship, with significant causality running from ACF to trade (p < 0.0001) but not vice versa, indicating that agricultural production patterns may influence trade structures more significantly than trade liberalization affects agricultural emissions in these economies.

Particularly noteworthy is the strong unidirectional causality from GDP per capita to trade openness (p < 0.0001), with weaker causality in the opposite direction (p < 0.05), suggesting that economic development primarily drives trade integration rather than trade driving growth in this sample—a finding that offers important nuance to conventional trade-led growth narratives but aligns with development trajectories of several Asian economies where strategic industrial policies often preceded deep trade liberalization. The finding regarding strong unidirectional causality from GDP per capita to agricultural carbon footprints align with Cui et al.'s^20^ identification of an environmental Kuznets curve in agricultural greenhouse gas emissions in China’s principal grain-planting regions, suggesting that economic development fundamentally reshapes agricultural emission profiles.

Similarly, our result complements Khan and Yahong^39^ verification of "a beneficial correlation between disparities in earnings, environmental impact, and greenhouse gases" across 18 Asian emerging nations. However, our causality analysis provides stronger directional evidence than these previous studies, clearly establishing that economic development drives agricultural emission patterns rather than the reverse.

The Dumitrescu-Hurlin country-specific causality test results is reported in Table 11. The findings reveal significant heterogeneity in the causal relationship between agricultural carbon footprints and sustainable development across the nine sampled economies. China and India demonstrate very strong evidence of Granger causality from ACF to SD (p < 0.01), reflecting the outsized importance of agricultural activities in these two populous nations where agriculture remains a significant economic component despite rapid industrialization.

**Table 11:** Dumitrescu & Hurlin (2012) Granger non-causality test results (Country Specific).

Country	Wi	PVi
Indonesia	1.5202	0.4837
Viet Nam	3.0314	0.2495
India	13.144	0.0082
Malaysia	3.8852	0.1756
Philippines	6.8685	0.0574
Bangladesh	0.3127	0.8565
China	15.726	0.0041
Thailand	3.9544	0.1708
Mongolia	3.7410	0.1862

Source: Authors’ compilation (2024).

Note: H0: lnACF does not Granger-cause lnSD; H1: lnACF does Granger-cause lnSD).

The Philippines shows moderate evidence of causality (p < 0.10), while the remaining countries—Indonesia, Vietnam, Malaysia, Bangladesh, Thailand, and Mongolia—do not exhibit statistically significant causal relationships at conventional levels. This heterogeneity parallels Sun et al.'s^64^ observation that the impact of poverty elimination programs on greenhouse gas emissions varies dramatically across Chinese provinces, with emissions growth potentially "reaching 4.0% in developing areas, nearly five times greater than in prosperous areas.

Similarly, our findings reinforce Du et al*.’s*^24^ conclusion that the effectiveness of agricultural sustainability policies varies significantly across Chinese prefectures depending on local economic conditions. By demonstrating similarly stark variations in causal relationships across different Asian economies, our results extend this heterogeneity insight beyond China to the broader Asian context—underscoring the critical importance of nationally tailored approaches to agricultural sustainability that account for country-specific causal mechanisms.

Recent studies reaffirm the complex nexus between agricultural carbon footprints, renewable energy, and sustainable development. Anser et al.^73^ highlighted the environmental impact of shadow economies, while Xu et al.^74^ demonstrated technological interventions reducing emissions. Lin et al.^75^ , Teron et al.^76^ ; Shang and Luo^77^ emphasized sustainable agricultural practices and biophysical factors in carbon mitigation. Luan et al.^78^ and Osabohien et al.^79,80^ found renewable energy promotes sustainability but noted implementation challenges. Guo et al.^81^ underscored institutional quality’s moderating role in resource-sustainability dynamics. These findings align with our study, confirming that agricultural emissions, though detrimental, can contribute positively to development when managed through targeted policies, technological adoption, strong institutions and food security, in line with Ramankutty et al. ^82^.

## Conclusion and policy recommendations

This study examined the relationships among agricultural carbon footprints (ACF), renewable energy consumption (RE), and sustainable development (SD) in nine Asian nations from 2000 to 2022. The study employed Cross-Sectional Autoregressive Distributed Lag (CS-ARDL), Method of Moments Quantile Regression (MM-QR), and Dumitrescu-Hurlin panel (DH) causality techniques to examine the nexus. The findings of the Dumitrescu-Hurlin panel (DH) causality demonstrated significant bidirectional causality between sustainable development and agricultural carbon footprints, hence, exhibiting notable heterogeneity among countries—especially pronounced in China and India.

The MM-QR analysis revealed that agricultural activities significantly enhance sustainable development in lower-performing countries; however, this beneficial correlation decreases at elevated sustainability levels, indicating stage-dependent dynamics. While renewable energy usage exhibits bidirectional causation with sustainable development, its direct effect is statistically insignificant across all quantiles, suggesting implementation constraints that hinder Asian economies from fully exploiting clean energy potential.

Firstly, our findings require distinct agricultural policy strategies customised to the sustainable development stages of each country. Less advanced economies should implement policies that optimise the socioeconomic advantages of agriculture while progressively adopting sustainable practices; conversely, more developed economies should concentrate on diminishing carbon intensity without sacrificing productivity. Despite limited statistical significance, the bidirectional causality between renewable energy and sustainable development suggests unrealized potential that requires addressing implementation barriers, increasing infrastructure investment, and developing coherent policy frameworks. The consistently negative relationship between trade openness and sustainable development demands reform, including stronger environmental provisions in trade agreements, support for sustainable export industries, and protective measures for local communities.

Second, the differences in the DH country-specific results underline the need of enhancing governance structures and using comprehensive policies that handle the complicated interaction between agricultural production, energy consumption, economic development, and environmental results. Our results expose that the road towards sustainability vary significantly depending on economic situation and development level, therefore challenging the oversimplified stories sometimes seen in policy debates. Countries seem to follow different paths impacted by their own economic systems, resource endowments, and institutional frameworks instead of a homogeneous trajectory. Beyond limited technical fixes, sustainable growth in these Asian nations will call for integrated strategies balancing social and economic goals with environmental factors. This includes realising how the contributions of agriculture and renewable energy to sustainability outcomes change as economies grow and building trade systems that more closely match economic integration with social equality and environmental protection.

The limits of this study lie in the data constraints within the 2000–2022 period which may not fully capture dynamics of long-term sustainability, aggregation issues that possibly obscure differences between agricultural subsectors, as well as limited country coverage. Future studies are implored to pursue disaggregated analysis of agricultural carbon footprints by subsector, expand country coverage, incorporate institutional quality measures, examine distributional impacts across socioeconomic groups, and develop more robust approaches to measuring sustainable development that better reflect local priorities.

## Data Availability

Data will be made available by the responding author upon request. Therefore, the corresponding author should be contacted to request the data from this study.
